# Unravelling the therapeutic potential of IL-33 for atrophic AMD

**DOI:** 10.1038/s41433-021-01725-5

**Published:** 2021-09-16

**Authors:** Alison J. Clare, Jian Liu, David A. Copland, Sofia Theodoropoulou, Andrew D. Dick

**Affiliations:** 1 Academic Unit of Ophthalmology, Translational Health Sciences, University of Bristol, Bristol, UK; 2 School of Cellular and Molecular Medicine, University of Bristol, Bristol, UK; 3 NIHR Biomedical Research Centre of Ophthalmology, Moorfields Eye Hospital, London, UK; 4 UCL Institute of Ophthalmology, London, UK

**Keywords:** Macular degeneration, Immunotherapy

## Abstract

Age-related macular degeneration (AMD), a degenerative disease affecting the retinal pigment epithelium (RPE) and photoreceptors in the macula, is the leading cause of central blindness in the elderly. AMD progresses to advanced stages of the disease, atrophic AMD (aAMD), or in 15% of cases “wet” or neovascular AMD (nAMD), associated with substantial vision loss. Whilst there has been advancement in therapies treating nAMD, to date, there are no licenced effective treatments for the 85% affected by aAMD, with disease managed by changes to diet, vitamin supplements, and regular monitoring. AMD has a complex pathogenesis, involving highly integrated and common age-related disease pathways, including dysregulated complement/inflammation, impaired autophagy, and oxidative stress. The intricacy of AMD pathogenesis makes therapeutic development challenging and identifying a target that combats the converging disease pathways is essential to provide a globally effective treatment. Interleukin-33 is a cytokine, classically known for the proinflammatory role it plays in allergic disease. Recent evidence across degenerative and inflammatory disease conditions reveals a diverse immune-modulatory role for IL-33, with promising therapeutic potential. Here, we will review IL-33 function in disease and discuss the future potential for this homeostatic cytokine in treating AMD.

## 摘要

年龄相关性黄斑变性(AMD)为影响黄斑区视网膜色素上皮(RPE)和光感受器的退行性疾病, 是老年人中心性视力丧失的主要原因。当AMD进展到晚期, 例如萎缩性AMD (aAMD)或15%的“湿性”或新生血管性AMD (nAMD), 将伴有严重的视力丧失。虽然nAMD的治疗手段有了进步, 但迄今为止, 对于85%的aAMD患者, 尚没有有效的疗法, 只能通过改变饮食、补充维生素和定期监测来控制疾病。AMD的发病机制复杂, 涉及高度整合以及常见的年龄相关的疾病通路, 包括补体/炎症失调、自噬受损和氧化应激。AMD发病机制的复杂性使得治疗的发展具有挑战性, 确定一个与疾病融合的靶点对于提供全球有效的治疗是至关重要的。白细胞介素−33是一种细胞因子, 在过敏性疾病中起促炎作用。最近有关退行性疾病和炎症性疾病的证据显示, IL-33具有多种免疫调节作用, 具有很好的治疗潜力。在这里, 我们将回顾IL-33在疾病中的作用, 并讨论这种稳态细胞因子治疗AMD的未来潜力。

## Introduction

Age-related macular degeneration (AMD) is the leading cause of blindness among over-65s in developed countries and accounts for 8.7% of all blindness worldwide [1, 2]. With an ever-growing aging population, AMD has become an increasing health problem. Characterised by the progressive loss in the macular region of specialised cells, retinal pigment epithelium (RPE), and photoreceptors, AMD can progress into two advanced forms the “neovascular”-wet form (nAMD) and for the majority of patients the “atrophic”-dry form (aAMD). Whilst later stages of nAMD can, to various degrees, be successfully treated with VEGF-blocking agents, treatments to target early in the disease or tackle advancement to neovascularisation or late-stage aAMD are still lacking [2–6]. With the disease projected to affect 288 million people globally by 2040 [1], the unmet need for an effective therapeutic in early AMD is pressing.

Age is the leading risk factor for AMD and the aetiology is multifactorial. Data implicates the presence of chronic low-grade inflammation in the pathogenesis of AMD but the underlying causes and mechanisms responsible for persistent inflammatory responses are not fully appreciated. Environmental risk factors, including diet, obesity, light exposure, and smoking, alongside susceptibility genes, increase the risk of developing and are associated with greater severity of disease [7]. Multiple genome-wide association studies have identified risk loci for AMD. For example, strong associations are seen with complement pathway and ARMS2 alleles, which denote a large increase in disease risk, and identification of very rare coding variants in *CFI*, *CFH*, and *TIMP3* suggests causal roles for these genes [8, 9]. A more recent study has teased out risk variants in early vs. late AMD, identifying variants in pathways affecting complement complex and lipid metabolism as significant contributors in early and advanced AMD, with extracellular matrix metabolism identified among advanced-AMD risk loci only [10]. Understanding of genetic risk has driven approaches to developing therapeutics, including inhibitors and gene therapies targeting complement pathway [11–13]. However, the low penetrance of such genetic risk factors [8, 9] has led to mixed success with the greatest promise seen only for the small number of patients with relevant risk variants [11]. The disease complexity and involvement of converging pathways are supported as mice with complement perturbation do not fully emulate AMD pathology [14–16]. In addition to immune-mediated inflammation, pathways implicated in AMD include oxidative stress, mitochondrial dysfunction, lipid metabolism, autophagy, and cellular senescence [4–6, 17], all of which are interrelated and exert a cause-and-effect on each other; a vicious cycle ultimately leading to retinal degeneration. These immune, metabolic, and tissue responses occurring in AMD and retinal degeneration involve the integrated function of multiple cell types (such as RPE, microglia, choroidal endothelial, macrophages, and mast cells as well as infiltrating immune cells), and a combination of several genetic and environmental factors [2, 5, 6, 18]. Targeting therapeutic approaches that modulate cellular biogenetics and immune pathways may serve to protect the normal cell and tissue health, reaching a wider patient population.

The early stages of AMD are clinically defined by the presence of small drusen deposits and pathological changes to the RPE [19]. Located and notwithstanding presence of subretinal drusenoid deposits between the Bruch’s membrane and photoreceptors, the RPE is central in the maintenance of the visual cycle, and photoreceptor health [17, 19]. As such, RPE is metabolically demanding and mitochondria rich [20]. Through high-turnover phagocytosis of the outer segment and photo-stress, RPE mitochondrial respiration serves a major source of endogenous reactive oxygen species (ROS), which in turn and with age may lead to damage of mitochondria and the cellular integrity [17, 20]. Indeed, metabolomics analyses of RPE from human AMD donors demonstrate significantly aberrant mitochondrial metabolites [21]. Whilst healthy RPE rely on antioxidative defences, including autophagy/mitophagy [20], iPS-RPE-derived cells from AMD patients, or with known risk mutations have reduced antioxidative stress response and produce higher levels of ROS [22, 23]. Defective RPE metabolism that associates with a low-grade unresolved inflammation, is considered as a major trigger event for AMD progression.

Metabolic dysfunction and oxidative stress are highly integrated with immune function within the tissue microenvironment (metabolic ecosystem) and in individual cells [5, 19, 20, 24, 25]. Excessive inflammatory mediators, such as cytokines, ROS, and nitric oxide, undermine mitochondrial function by inducing mitochondria DNA (mtDNA) mutations and inhibiting the mitochondrial respiratory chain for energy production [6, 17]. Concurrently, lipids, proteins, and DNA damaged by oxidation found in drusen and RPE are proinflammatory [20, 26]. These tissue stressors accumulate with age and induce a para-inflammatory response [27], which plays a protective role for the retina, for example driving phagocytosis of the drusen [28]. However, in AMD, a notion is that homeostatic para-inflammation itself becomes dysregulated and drives detrimental inflammation possibly due to excessive damage (e.g. from age or environmental factors) or genetic perturbation affecting the normal immune response (e.g. complement pathway risk alleles) [27, 29]. In this instance, a host of proinflammatory cytokines secreted from RPE, microglia, and macrophages, along with degranulation of choroidal mast cells [30–32] promote a proatrophic and proangiogenic environment for AMD progression [5, 33–35]. Overcoming such immune dysregulation, is a key therapeutic target. For example, eliciting conditions that promote a regulatory proresolution inflammatory phenotype has been shown to be antiangiogenic in experimental autoimmune uveitis and laser-induced CNV, respectively [5, 36, 37], and could have anti-inflammatory potential in AMD. Moreover, whilst diverse immune regulators, such as IL-10 and IL-4 are central for the immune homeostasis; there is increasing evidence that manifests their indispensable roles in also regulating cellular metabolism and maintaining cell health [38, 39]. Indeed, metabolic programming of cells is distinct depending on the immune stimulator with IL-4 inducing an M2 skew in macrophages and commitment to oxidative phosphorylation (OXPHOS), whilst lipopolysaccharide (LPS) promotes a switch to aerobic glycolysis [40]. Moreover, metabolism and metabolites can directly influence the function and differentiation of immune cells (reviewed in [40, 41]). In this review, we will cover the immunomodulatory potential of homeostatic cytokine interleukin-33 (IL-33), and the growing evidence supporting its therapeutic potential to target aspects of early metabolic and immune dysregulation in AMD.

## Interleukin-33 modulation of disease

IL-33 is a nuclear cytokine of the IL-1 family, which is constitutively and strongly expressed in epithelial, endothelial, and fibroblasts of barrier tissues [42–44]. IL-33 is considered an alarm signal (“alarmin”), a first-line defence responding to both endogenous and exogenous danger signals, such as tissue damage or allergen exposure [44, 45]. However, constitutive IL-33 expression in cell types, such as adipocytes, glial cells, and neurons [44, 46–48], suggest a broader role for this cytokine beyond alarmin inflammatory function, highlighting potential roles in cellular and tissue homeostasis. Unlike the other IL-1 cytokine family members, IL-33 lacks a classical signal peptide for secretion from cells and is bioactive at full length. Thus, the effect of a “surge” IL-33 release from cells is best understood in conditions of allergy, necrosis, or mechanical injury [42]. Although, IL-33 is also secreted without cell death, in response to cell stressors, such as oxidative stress or increase of extracellular ATP [49–51], the mechanism of release remains elusive. Once secreted, IL-33 activity can be enhanced by proteases, in the context of acute or subacute inflammatory responses, from immune cells or allergens [42] or suppressed by soluble ST2 (decoy receptor) [52, 53] and caspase-dependent proteolysis [54]. Signalling via the ST2 receptor (IL-1RL1R) [42], IL-33 has a wide range of cellular targets, including innate lymphoid cells 2 (ILC2s) [42, 55], mast cells [56], T-helper (Th)2 cells [42], macrophages [57], eosinophils as well all Th1, and natural killer cells [42, 58]. As such, IL-33 plays a critical immune-modulatory role in allergic and chronic inflammatory and degenerative diseases, from asthma and cardiovascular disease to Alzheimer’s disease [44, 59–61].

As an ”alarmin” and strong inducer of Th2 immune responses, IL-33 is implicated in Th2-mediated allergic inflammatory disorders, such as asthma [44]. In lung biopsies of asthmatic patients, IL-33 expression is increased [62] and IL-33 polymorphisms can increase asthma susceptibility [63, 64]. This is supported by observations in mice, where overexpression or exogenous administration of IL-33 promotes airway inflammation, whilst blocking the IL-33-ST2 axis reduces inflammation [44, 65]. In this context, IL-33-ST2 signalling mediates both innate and adaptive Th2 responses in asthma, including eosinophils, ICL2s, Th2 cells, and mast cells [44], inducing secretion of proinflammatory cytokines and cellular degranulation. However, IL-33-stimulated mast cells are also important for balancing immune homeostasis in models of asthma, promoting the expansion of Treg cells [66], and protecting against the development of airway hyperresponsiveness [67]. Given these opposing regulatory roles, it may be the aberrant expression and activity of IL-33, exacerbated by allergens [42], that promotes dysregulation and subsequent pathological chronic inflammation. Certainly, under acute phototoxic stress in the retina, Müller glia cells secreted supraphysiological amounts of IL-33 into the vitreous leading to cell death and retina thinning [46]. Conversely, in the absence of provoking an acute release with or without aggravating exogenous environmental cues, IL-33 can adopt an important protective role, modulating the immune response and resolving inflammation in metabolic and autoimmune disorders, as well as mechanical injury [36, 57, 68–70].

**Fig. 1:**
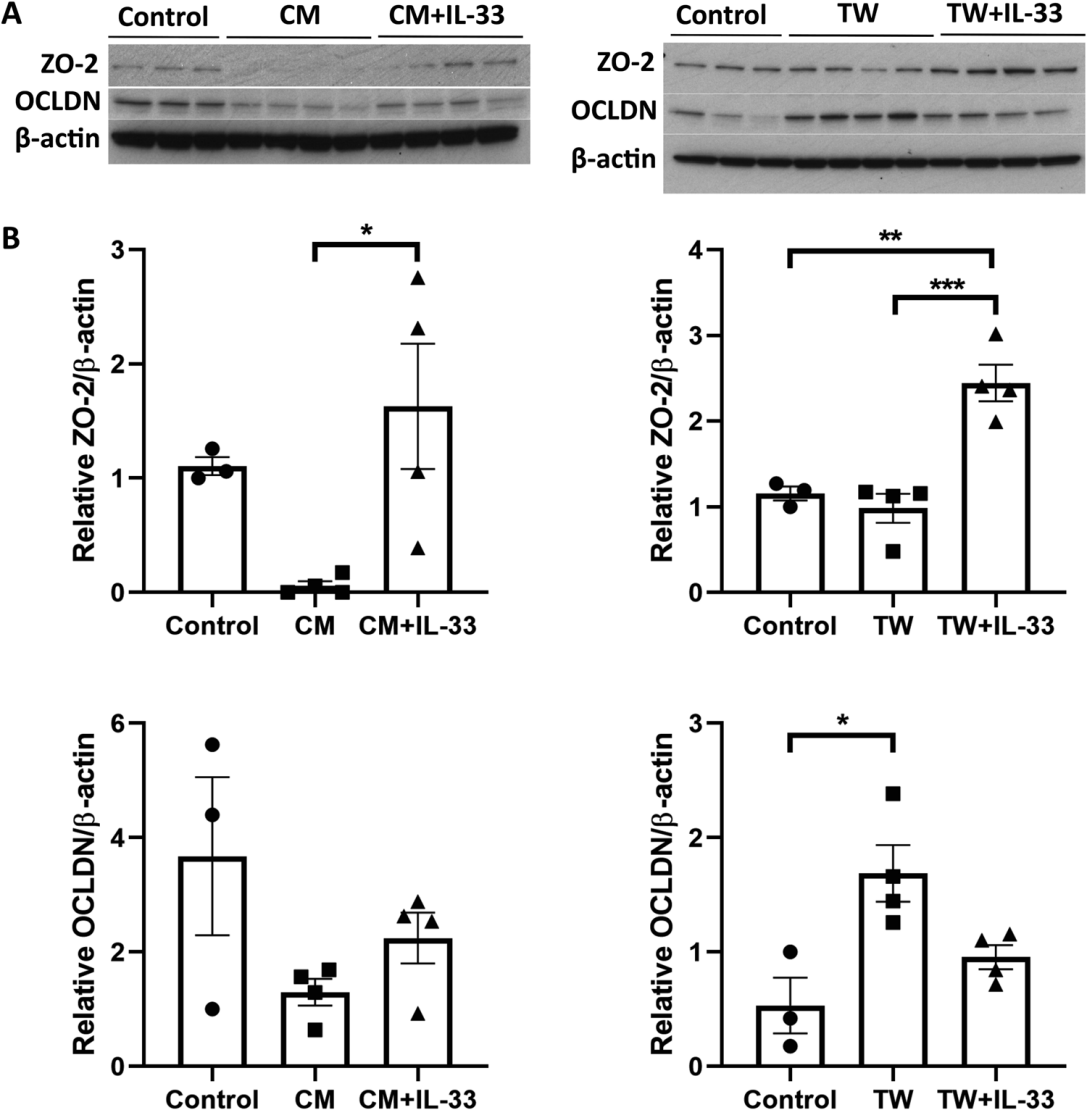
IL-33 stimulated mast cells regulate tight-junction protein expression in RPE. **A** Western blot shows ZO-2, Occludin and beta-actin expression in B6RPE-07 lysates (mouse RPE cell line, courtesy of H Xu, Belfast) either cultured in standard media (control), in conditioned media (CM) from bone-marrow-derived mast cells with or without IL-33 stimulation (12 h), or co-cultured by transwell (TW) with treated or untreated mast cells. **B** Graphs show relative expression by densitometry analysis (*p < 0.05, **p < 0.01; one-way ANOVA with Tukey’s multiple comparisons test).

Patients with chronic heart failure have a reduced IL-33/sST2 ratio, suggesting an importance for IL-33 bioactivity, which is hindered by inhibitory sST2 [71]. Indeed, in mice, IL-33-mediated expansion of IL-5 and −13 expressing Th2 cells protects against atherosclerosis by stimulating the production of ox-LDL antibodies [59]. Similarly, in diet-induced obese mice, IL-33 expansion of ST2+ Treg cells reduces inflammation in visceral adipose tissue ameliorating insulin resistance [70], and in the eye retinal inflammation in the experimental autoimmune uveitis (EAU) model is attenuated by IL-33 expansion of Th2 cells and reduction of pathogenic Th17 inflammatory cells [36]. Interestingly, in the experimental autoimmune encephalomyelitis (EAE) model IL-33 can attenuate expansion of Th17 cells through mast cell-mediated alternative activation of macrophages (M2 skew), suggesting mast cells could play an important intermediary role in mediating protective effects of IL-33 [72]. In the eye, we have demonstrated that infiltration of tryptase+ mast cells at lesion sites in the laser-induced choroidal neovascularisation (L-CNV) model, similar with both vehicle and IL-33 treatment [73]. Furthermore, co-culture with bone-marrow-derived mast cells or the conditioned media from the cells had a significant effect on the RPE cell expression of tight-junction proteins, zona occludins 2 (ZO-2), and occludin (OCLDN), when primed with IL-33 compared to unstimulated cells (Fig. 1). Changes to tight-junction proteins, including ZO-2 and OCLDN, are important in wound-healing responses and RPE barrier integrity and function [74], and therefore the IL-33-mediated response of mast cell function could influence the outcome in this context. Furthermore, the importance of IL-33 in resolving inflammation is highlighted in a study by Augustine et al. that shows following retinal detachment, IL-33 deficient mice display chronic inflammatory responses and increased severity of retinal degeneration [68]. As in EAE, IL-33-mediated alternative activation of macrophages also plays an important role in promoting recovery after a spinal cord injury [57]. Whilst in the brain, IL-33-stimulated macrophage M2 phenotype of microglia is both key to maintaining synaptic plasticity, protecting against cognitive decline [47], as well as stimulating clearance of plaques in Alzheimer’s models [60, 75]. The importance of IL-33 in modulating immune responses across diseases settings, highlights the homeostatic potential for this cytokine. However, the broad constitutive expression of IL-33 across cell types, including neurons, also suggests a wider role outside of immune regulation.

In brain tissue from Alzheimer’s patients, IL-33 expression is decreased, and overexpression of nuclear IL-33 in vitro reduces the secretion of Amyloid-beta (Aβ) [40] peptides, independent of ST2 signalling [76]. Whilst it is recognised that IL-33 stimulation of macrophages is important for clearing Aβ plaques [60, 75], other reports demonstrate that IL-33 loss impairs the autophagic clearance of cell waste in neurons and leads to furthers neuronal loss [48]. In ovarian tissue, loss of IL-33 resulted in the loss of autophagic function, subsequent waste accumulation and tissue degeneration [77]. Together, these data strongly infer a critical role for IL-33 in maintaining autophagy and thus cell heath and tissue function across a variety of tissues.

IL-33 stimulation also has a direct effect on cell metabolism, increasing glycolysis and OXPHOS in mast cells [78] and reshaping metabolism in macrophages [79]. Our recent work further uncovered IL-33/ST2-mediated changes to metabolism in RPE cells but importantly revealed a novel role for nuclear IL-33 as a critical metabolic regulator (discussed in the next section) [80]. Thus, these findings and emerging evidence supports a broader role for IL-33, through both the IL-33/ST2 signalling axis and within the nucleus. Moreover, with a homeostatic role in immune modulation, metabolism and autophagy, the therapeutic potential for IL-33 is extensive and far reaching, in particular, for tissues with a high metabolic load, such as the CNS and retina.

## Therapeutic development and the potential of IL-33 for treating aAMD

The complexity of AMD pathogenesis has led to several approaches for therapeutic development, targeting the different pathways that contribute to disease progression. The discovery of complement associated risk variants combined with the identification of complement components in drusen [81], has driven a multitude of the investigation of complement pathway inhibitors as potential AMD therapeutics, with mixed results [82]. Many approaches investigated follow a paradigm of intravitreal injections, which still impacts patient compliance and ability to deliver at scale in healthcare settings. As such, complement inhibition and anti-inflammatory therapies are expanding into gene therapy approaches. The pipeline of gene therapy for ophthalmic eye disorders is expanding, with successes in inherited retinal degenerative disorders [83] and the potential for a “one-hit” treatment approach for AMD may provide great appeal.

Due to the importance of RPE in maintaining the retina and photoreceptor health and the contribution of RPE oxidative stress to pathological progression of AMD, another therapeutic strategy is antioxidant supplement treatment and mitochondrial enhancer, Elamipretide, with some success in early trials [82]. Certainly, the potential for gene augmentation of oxidative stress modulators is being investigated further and bring an opportunity to drive additional candidate targets for gene therapy in AMD. The push towards identifying gene therapy targets for AMD is a promising avenue for treatment, however; due to AMD complexity identifying the right candidate is essential for reaching the wider aAMD patient population. Considering the convergence of affected pathways, autophagy/mitophagy, oxidative stress/metabolism and inflammation, the efficacy of gene therapy in AMD will be maximised by the identification of a cross-regulatory target.

**Fig. 2:**
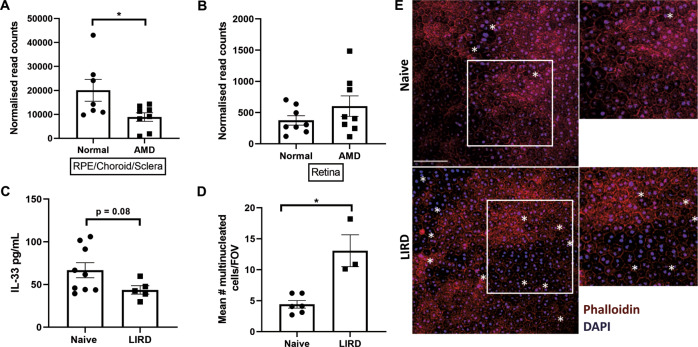
Association of altered IL-33 expression with AMD and RPE damage. IL-33 gene expression is decreased in RPE/choroid/sclera of AMD donors by more than 50% compared to normal age-matched donors (p = 0.03, two-tailed t-test) **A** but expression is unchanged in the retina **B**. RNA- sequencing data from Kim et al. (GSE99248). **C** ELISA analysis shows a reduction of IL-33 protein in RPE lysates from a mouse model of light-induced retinal damage (100k LUK/20 min), compared to naïve eyes (*p* = 0.08, Mann–Whitney *U* test). **D** Concurrently, RPE damage is apparent, indicated by an increase in the number of multinucleated cells (RPE cells containing ≥3 nuclei/cell within the cell boundary; *p = 0.02, Mann–Whitney *U* test), counted using ImageJ. **E** Representative images of RPE/choroid flatmounts, stained with phalloidin-Alexa555 and DAPI. Multinucleated cells are indicated (*). Scale bar is 80 μm.

In our recent work, we illuminated a novel role for nuclear expressed IL-33 as a key regulator of metabolism in RPE cells. The nuclear expression of IL-33 is essential for utilisation of pyruvate in the TCA cycle and mitochondrial metabolism, through inducing the abundance of mitochondrial pyruvate carrier 1 (MPC1). Primary RPE cells from IL-33-/- mice have significant changes to mitochondria structure compared to wild-type cells, displaying irregularity in size and increased short tubular phenotypes [80]. Similar mitochondrial changes were observed in human RPE cells from donors >60 years in age compared to young donors and correlated with a reduction in antioxidant defences [84] and IL-33 expression (see below), consolidating the importance of IL-33 expression for mitochondrial structure and metabolism. Conversely, IL-33 antagonises aerobic glycolysis, so that with loss of IL-33 expression cells exhibit increased extracellular acidification rate (ECAR) and extracellular lactate [80]. Reduction or loss of IL-33 in RPE could lead to devastating changes to the outer retinal homeostasis, particularly if RPE adopt aerobic glycolysis over mitochondria respiration [85, 86] and yield RPE vulnerable to oxidative stress, conditions permissive for AMD pathogenesis. Indeed, the data-mining approach of published transcriptomic datasets reveals that the RPE/choroid/sclera tissues of AMD patients have decreased IL-33 mRNA expression compared to normal age-matched donors, whilst expression is unchanged in the retina (Fig. 2A, B (ref [87])). In support, and specific to the RPE, we see a reduction in IL-33 protein expression and concurrent RPE damage in a mouse model of light-induced retinal damage, emulating oxidative stress (unpublished, Fig. 2C–E). The nuclear importance of IL-33 to the RPE metabolic phenotype is distinct from the IL-33/ST2 signalling, as evidenced by the lack of metabolic phenotype in IL1rl1-/- mice [80]. However, in parallel we find IL-33 signalling via ST2 also contributes to promoting oxidative phosphorylation and mitochondrial respiration in RPE cells, through activation of AMP-activated protein kinase (AMPK). Importantly, treatment of RPE cells with recombinant IL-33 protects against H_2_O_2_ induced oxidative stress [80], suggesting IL-33/ST2 signalling could have therapeutic potential for RPE in AMD. Accordingly, we have shown IL-33 delivery in the aged *Cfh*+/− high-fat diet-fed model of outer retinal degeneration can protect against RPE loss and maintain expression of key metabolic proteins in the retina [88]. Additional evidence shows that stimulation of AMPK by metformin protects against photoreceptor and RPE damage in retinal degeneration models, including light-induced damage [89], and that activation of AMPK converges on pathways promoting autophagy [90]. IL-33 signalling could therefore also have beneficial effects for autophagy in RPE cells, another key target for combating pathogenic changes in AMD. Loss of IL-33 in the brain and ovarian follicle tissues leads to a reduction in a central autophagy protein, LC3II, supporting an important role for IL-33 in maintaining autophagy [48, 77]. Collectively, through distinct mechanisms, IL-33 has potential both as a nuclear effector and by canonical IL-33/ST2 signalling to maintain and promote metabolic homeostasis and autophagy in RPE. Therapeutically targeting converging metabolic and autophagic pathways brings an opportunity to ameliorate disease progression in the early stages of aAMD.

**Fig. 3:**
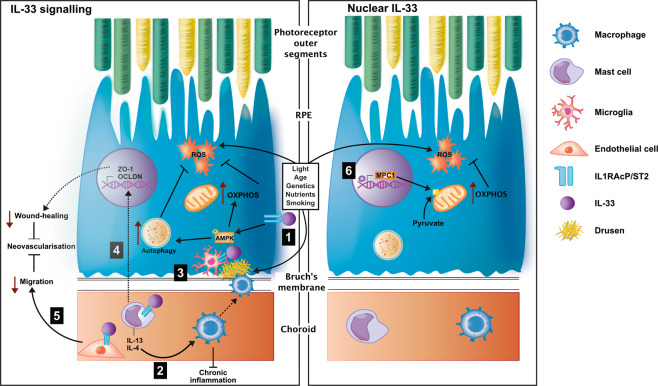
IL-33 therapeutic potential in early AMD. The interleukin-33 biological function has The potential to protect against early pathogenic pathways in AMD via dual function, as a signalling molecule and nuclear factor. With diverse cellular targets, IL-33 can stimulate 1) retinal pigment epithelium (RPE) in the eye directly, increasing AMPK phosphorylation and subsequent mitochondrial metabolism^78^. AMPK phosphorylation can also increase autophagy. Both will combat reactive oxygen species (ROS) production and oxidative stress. 2) IL-33 influences alternative activation of macrophages by stimulation of mast cells to release IL-13 and IL-4^68^, which can promote inflammation resolution. 3) IL-33 stimulation of an alternative microglia phenotype promotes deposit clearance in Alzheimer’s disease models^59,73^ and could prove beneficial against drusen. 4) IL-33 stimulated mast cells to induce changes to tight-junction proteins in RPE cells 5) and directly stimulate retinal endothelial cells reducing migration^71^, both important in mediating wound-healing responses. 6) Nuclear IL-33 is a key metabolic regulator in RPE cells, promoting increased mitochondrial metabolism (OXPHOS; oxidative phosphorylation)^78^. The dashed line indicates indirect evidence.

Further, in 10–15% of cases, AMD will progress to neovascularisation, driven by aberrant angiogenesis and fibrosis [91]. We have demonstrated that treatment of human choroidal fibroblasts with recombinant IL-33 reduces expression of MMPs (2/9), stalling cell migration, and the wound-healing responses behind fibrosis. Additionally, in a mouse model of choroidal neovascularisation, we found IL-33/ST2 signalling from recombinant IL-33 is antiangiogenic and reduces neovascular lesion volume [73]. Treating early in AMD with IL-33 could therefore not only be beneficial in targeting disease pathways of aAMD but also provide protection against advancement to neovascularisation. Together, evidence suggests IL-33 treatment early in AMD may protect RPE, promote metabolic and autophagic homeostasis, and modulate infiltrating cells, resolving pathogenic immune dysregulation and angiogenesis (Fig. 3).

## Conclusions

The complex multifactorial nature of early and aAMD pathogenesis provides a huge challenge in developing effective therapeutics to benefit a wide population of patients. Advancements in understanding the disease pathways and risk alleles behind AMD has supported the development of several targeted therapeutic approaches. However, identifying a target that can tackle multiple pathways of aAMD pathogenesis remains critical. Growing evidence of the homeostatic nature for cytokine IL-33, mediating metabolism, autophagy, and modulating the immune response, provides the exciting potential to target the converging disease pathways of AMD. Future work in models emulating disease pathways of aAMD will be important to confirm beneficial effects for IL-33 in modulating immune dysregulation under disease conditions.
